# UFCG: database of universal fungal core genes and pipeline for genome-wide phylogenetic analysis of fungi

**DOI:** 10.1093/nar/gkac894

**Published:** 2022-10-22

**Authors:** Dongwook Kim, Cameron L M Gilchrist, Jongsik Chun, Martin Steinegger

**Affiliations:** Interdisciplinary Program in Bioinformatics, Seoul National University, Seoul 08826, Republic of Korea; School of Biological Sciences, Seoul National University, Seoul 08826, Republic of Korea; Interdisciplinary Program in Bioinformatics, Seoul National University, Seoul 08826, Republic of Korea; School of Biological Sciences, Seoul National University, Seoul 08826, Republic of Korea; Institute of Molecular Biology and Genetics, Seoul National University, Seoul 08826, Republic of Korea; Interdisciplinary Program in Bioinformatics, Seoul National University, Seoul 08826, Republic of Korea; School of Biological Sciences, Seoul National University, Seoul 08826, Republic of Korea; Institute of Molecular Biology and Genetics, Seoul National University, Seoul 08826, Republic of Korea; Artificial Intelligence Institute, Seoul National University, Seoul 08826, Republic of Korea

## Abstract

In phylogenomics the evolutionary relationship of organisms is studied by their genomic information. A common approach to phylogenomics is to extract related genes from each organism, build a multiple sequence alignment and then reconstruct evolution relations through a phylogenetic tree. Often a set of highly conserved genes occurring in single-copy, called core genes, are used for this analysis, as they allow efficient automation within a taxonomic clade. Here we introduce the Universal Fungal Core Genes (UFCG) database and pipeline for genome-wide phylogenetic analysis of fungi. The UFCG database consists of 61 curated fungal marker genes, including a novel set of 41 computationally derived core genes and 20 canonical genes derived from literature, as well as marker gene sequences extracted from publicly available fungal genomes. Furthermore, we provide an easy-to-use, fully automated and open-source pipeline for marker gene extraction, training and phylogenetic tree reconstruction. The UFCG pipeline can identify marker genes from genomic, proteomic and transcriptomic data, while producing phylogenies consistent with those previously reported, and is publicly available together with the UFCG database at https://ufcg.steineggerlab.com.

## INTRODUCTION

The taxonomic kingdom *Fungi* is one of the most diverse clades in the tree of life, potentially encompassing 2.2–3.8 million species (1). Publicly listed fungal species in the RefSeq database (2) have exponentially grown from only 157 to 16 869 species in the last 12 years. Fungal resources, such as available genomes or proteomes are growing rapidly and are powering the resolution of phylogenetic relationships within the clade.

Before this wealth of fungal resources became available, only few near universally present markers were used for phylogenetic analysis. The internal transcribed spacer (ITS) region of the nuclear ribosomal RNA (rRNA) cistron has long been used in phylogenetic analysis of fungi as the universal fungal marker (3–5) and has formed the basis of large-scale barcoding efforts (6,7). In cases where the ITS region does not provide adequate resolution, secondary markers may be used instead (8), such as RNA polymerases (9), translation elongation factors (10) and mitochondrial genes (11). The use of multi-gene phylogenies, where the ITS region is used in conjunction with these secondary markers, has become increasingly common to resolve taxonomic relationships (12–15). However, usage of secondary markers requires researchers to know which markers to select based on the lineage being studied, as well as how to practically extract, align, and concatenate them to infer their phylogenetic relationship (16).

With the rapid increase of available public genomic sequences analysis using multiple markers became feasible, which increased the resolution further (17). The most commonly used technique is to concatenate multiple genes that are single-copy and orthologous, while existing universally among the taxa (core genes; (18)). Core gene based automated phylogenomic pipelines have attained wide adoption in the prokaryotic kingdom, such as Genome Taxonomy Database (GTDB; (19)), AutoMLST (20) or UBCG (21).

There have been numerous efforts towards defining sets of single-copy orthologs for the fungal kingdom, notably the Fungal Genome Mapping Project (FGMP; (22)), and the Benchmarking Universal Single-Copy Orthologs pipeline (BUSCO; (23)), which implements OrthoDB datasets (24). However, the primary focus of these methods is on assessing the completeness of fungal genomes, and as such no single method integrates the entire process from core gene identification to phylogeny reconstruction.

**Figure 1.**
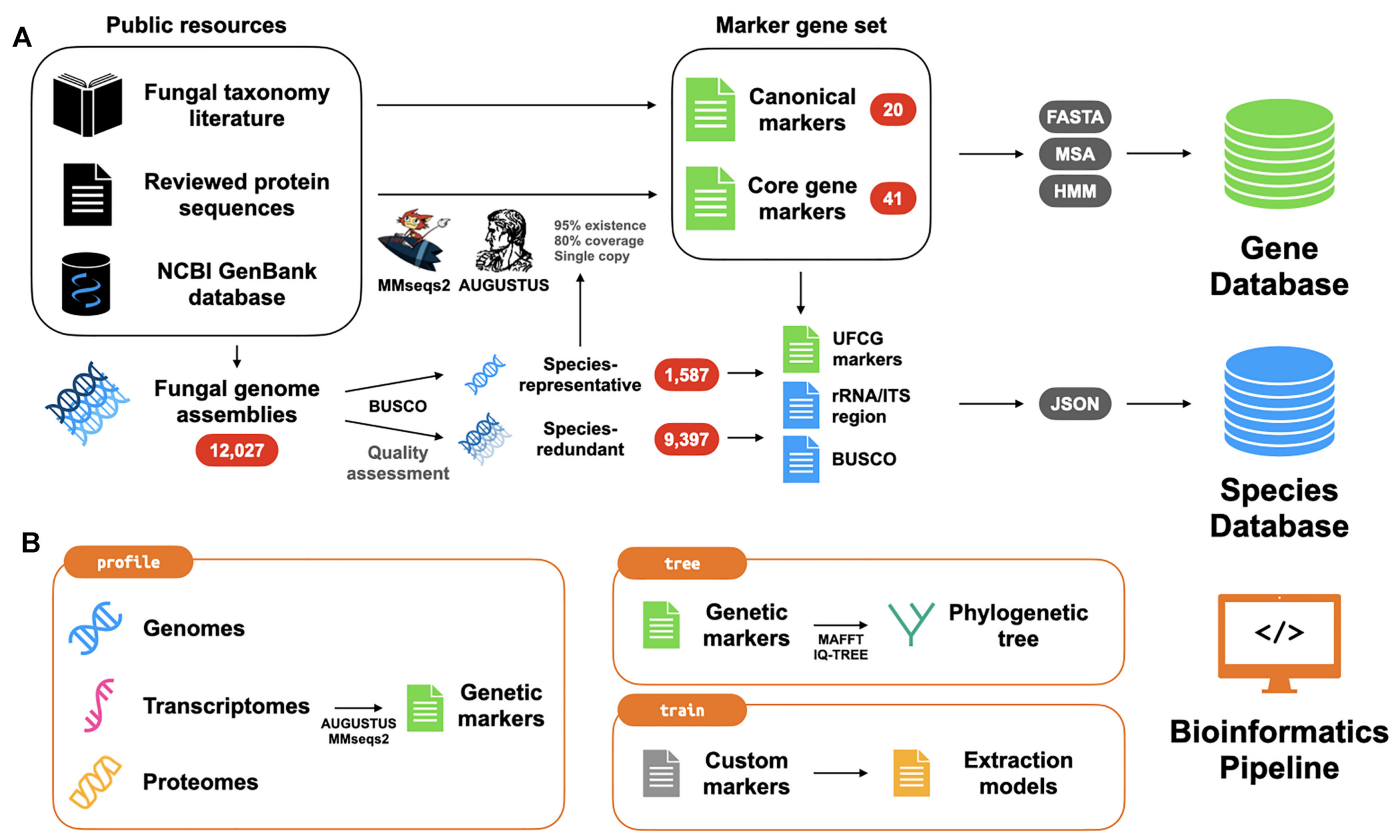
Schematic illustration of the preparation of the UFCG database and pipeline. (**A**) The UFCG Gene database consists of novel 41 core gene markers we defined, and 20 canonical marker genes curated from fungal taxonomy literature. We built profiles for all SwissProt Fungi proteins and searched them against 1587 species-representative genome assemblies using MMseqs2. Only genes that occur as single-copy in at least 95% species were further refined and filtered by AUGUSTUS-PPX. For each gene, we offer profile hidden Markov models (HMMs) and the seed amino acid sequences, downloadable from the database. The UFCG Species database provides pre-extracted marker sequences from the genome assemblies we obtained. In addition to the marker genes we defined, we extracted ITS and BUSCO sequences from both 1587 species-representative and 9397 species-redundant fungal genome assemblies. We compiled the extracted sequences into JSON files, which are downloadable from the database. (**B**) Graphical representation of three main modules (profile, tree, and train) from the pipeline. The profile module accepts genomic, proteomic, and transcriptomic data of fungi and extracts marker sequences using a pre-trained set of profile HMMs. The tree module combines the set of extracted marker genes and reconstructs their phylogeny as a maximum likelihood tree using aligned and concatenated marker sequences. The train module converts custom marker sequences into profile HMMs that can be directly utilized by the profile module.

Here, we introduce the Universal Fungal Core Genes (UFCG), a database of fungal marker genes derived from experimentally annotated genes (Figure 1A) as well as a pipeline for genome-wide phylogenetic analysis (Figure 1B). We defined 61 marker genes, 20 canonical markers extracted from literature research and 41 marker genes determined by computational identification of single-copy and highly conserved genes across the fungal tree of life, starting from manually curated and well-annotated sequences. The UFCG database provides freely accessible resources about the marker genes and fungal species from which the markers were identified, accompanied by a user-friendly website. We also provide an easy-to-use and fully integrated pipeline for fungal marker gene profiling and phylogenetics from fungal genomic, transcriptomic, and proteomic data.

## MATERIALS AND METHODS

### Preparation of genome assemblies

We obtained 12 027 whole genome assemblies of fungal species from the National Center of Biotechnology Information (NCBI) GenBank database (25) (Figure 1A). Taxonomic information from the NCBI taxonomy database (26) was assigned to each assembly. A single genome assembly was chosen for each fungal species by selecting those marked as representative in the GenBank database, excluding species with non-unique nomenclature (e.g. fungal *sp*.).

The completeness of the remaining assemblies was assessed by searching for single-copy orthologs using BUSCO v3.0.2 (27) with the OrthoDB v9 fungal lineage dataset (28). Additionally, we performed *ab initio* gene prediction using AUGUSTUS v3.4.0 (29) with a pre-trained species model of *Rhizopus oryzae*, which resulted in the highest average prediction count among the models (Supplementary Table S1). Assemblies that failed to report ≥250 BUSCO and ≥3000 predicted genes were removed from the set, resulting in 1587 species-representative assemblies.

### Core gene candidate detection with MMseqs2

For our core gene candidates we started with accurately annotated and experimentally validated genes from Swiss-Prot to avoid incorrectly called genes from contaminated genomic fragments (30) or fragmented genes because of limitations of eukaryotic gene finding software (31).

All 35 591 fungal protein sequences present in Swiss-Prot (release 2022_03; (32)), the manually curated part of the UniProt KnowledgeBase (33), were extracted and clustered to 90% sequence identity using MMseqs2 v13.45111 (34), resulting in 30 834 representative sequences. For each sequence, we generated a query centered multiple sequence alignment (MSA) by searching for three iterations (--num-iterations 3) against the full UniProtKB release 2022_03 (35) using MMseqs2. Each MSA was turned into a profile and searched against the species-representative assemblies using a MMseqs2 six-frame-translated sequence-to-profile search.

In some cases, hits to certain genes may be fragmented into multiple smaller hits (e.g. due to the intron-exon structure of eukaryotic genes), causing them to be filtered out in downstream analyses. In order to recover such genes, we implemented a procedure in which we merge hits to the same gene occurring sequentially on the same genomic contig. If the distance between the start position of the first hit and the end position of the final hit gives ≥80% query coverage, the merge was considered valid.

Genes that were identified as single-copy (i.e. only one valid hit discovered from the entire genome) from ≥95% of the 1587 species-representative assemblies were defined as candidate core genes, resulting in 62 genes.

### Profile HMM generation with AUGUSTUS

AUGUSTUS-PPX (36) provides a suite of scripts to generate block profile hidden Markov models (block profile HMMs; position-specific frequency matrices from a set of gap-less sequence blocks) from MSAs of homologous amino acid sequences, allowing sensitive and precise gene extraction from genome-scale data. We devised an iterative procedure using AUGUSTUS-PPX to build block profile HMMs with enriched homologous MSAs for 62 core gene candidates.

In each iteration, amino acid sequences of each gene are extracted from the species-representative assemblies with AUGUSTUS-PPX using the block profile HMMs from the previous iteration. Each extracted protein sequence was searched against the sequences from the respective MSA using MMseqs2. We accepted the protein sequence if its alignment covers at least 80% sequence length of a MSA member sequence. After each iteration a new MSA is generated by combining the previous and newly detected sequences using MAFFT v7.310 (37). The MSA is then used to build new block profile HMMs with AUGUSTUS-PPX.

For the first iteration, we used block profile HMMs built from the query centered MSAs (described above) for prediction and the amino acid sequences from Swiss-Prot with the corresponding gene annotation for homology search validation. We conducted three iterations of AUGUSTUS-PPX training for each of the core gene candidates we defined earlier, resulting in a final set of block profile HMMs.

### Quantifying the coverage of core gene candidates

To quantify the coverage of the core gene candidates on fungal species, we examined the presence of the genes from AUGUSTUS-PPX search against the species-representative genome assemblies. We repeated the final iteration of the profile HMM generation process described above to obtain the set of homologous protein sequences. The sequences with their alignment covering at least 80% sequence length of a member sequence of the respective MSA were accepted. A gene was defined present for the assemblies from which an accepted sequence was extracted.

For enhanced sensitivity, we relaxed the threshold and accepted the sequences covering at least 50% sequence length of a member. For the remaining sequences, the threshold was relaxed once more by accepting those aligned with *E*-value lower than 10^−3^.

We then tallied the proportion of the assemblies that reported the gene existing as a single-copy (i.e. only one homologous sequence detected), and those regardless of the copy number. Genes that ultimately failed to cover 95% of the species as a single-copy were rejected, while the remaining genes were defined as the final set of core marker genes.

To benchmark the core genes, we used the same method to quantify the existence coverage against 9397 species-redundant genome assemblies, which passed the quality assessment but were unused because of their taxonomic redundancy.

### Canonical marker genes

We found the absence of the conventional marker genes for multi-gene phylogeny from our computational investigation, due to their functional divergence and lack of universality across the entire kingdom (38,39). To supplement this, we collected a set of frequently used protein-coding phylogenetic markers from a review of fungal taxonomic literature, which we deemed canonical and included in the database. Profile HMM generation, coverage quantification, and benchmarking for these was performed identically as described for the core genes.

### Pipeline software development

We developed a bioinformatics pipeline integrating the process of marker gene extraction and phylogenetic analysis in a fully automated fashion. The modular pipeline allows users to process their biological sequences into sets of marker genes, align marker gene sequences, concatenate gene alignments, construct phylogenetic trees, and train their own marker MSAs and profile HMMs (Figure 1B).

We developed a pipeline with three main modules: profile, tree and train. The profile module accepts genome, transcriptome and proteome data as input, and extracts marker gene sequences with AUGUSTUS-PPX using pre-trained block profile HMMs. In addition to the UFCG markers, we prepared profile HMMs for the ITS region and 758 single-copy orthologs from the fungal subset of OrthoDB v10 (24) available for the module. The module validates the sequences with a MMseqs2 search against the pre-defined homologous sequences, with stepwise relaxation of thresholds (coverage ≥ 80%, coverage ≥ 50%, *E*-value < 10^−3^) as described above. A JSON file with valid amino acid and nucleotide sequences is produced as a result.

The tree module gathers the collection of JSON files produced with the profile module, constructs MSAs for each shared marker gene using MAFFT (37), removes alignment columns with a given gap threshold (default 50%), and generates phylogenetic trees in Newick format for the individual marker genes as well as from a concatenated MSA. For tree building, the user can choose among IQ-TREE (40), RAxML (41) and FastTree (42), with IQ-TREE being the default. Along with the bootstrap measure, the module computes a concatenation tree with branches annotated with Gene Support Index (GSI), the number of individual gene trees supporting the branch, as support values (21,43).

Finally, the train module fully automates the iterative profile HMM generation process described above. The module accepts seed marker sequences and reference genome assemblies, and generates profile HMMs that can be directly utilized by the profile module.

On average, the profile module takes 55 seconds to extract UFCG markers from a fungal genome with 32 CPU threads. The tree module requires 413 seconds to reconstruct a tree from 30 genomes with 32 threads using IQ-TREE v2.0.3. Detailed description and results of the runtime benchmark are summarized in Supplementary Table S2.

### Phylogenetic tree construction

To test our database and pipeline, we utilized the UFCG marker genes to reconstruct the phylogenetic trees of fungal lineages (Figure 3). Commands and parameters for the utilization of our pipeline are described in Supplementary Table S3.

First, to demonstrate the usability of our pipeline, we downloaded 34 sequence datasets from the order Eurotiales including 13 genomic, 8 transcriptomic and 13 proteomic sequences (Supplementary Table S4), and extracted their UFCG marker genes with the profile module of our pipeline. Marker gene extraction was performed with UFCG v1.0 profile module, which utilizes AUGUSTUS v3.4.0 and MMseqs2 v13.45111. The UFCG tree module automatically generated MSAs of the marker genes with MAFFT v7.310, removed alignment columns with ≥50% gaps, and drew the ML tree from the concatenation using JTT model (44) with IQ-TREE v2.0.3.

Additionally, we generated a kingdom-wide UFCG tree from genome assemblies of the entire 1587 fungal species (Supplementary Figure S2) to measure the phylogenetic consistency of our marker genes, with identical methods but using FastTree v2.1.10 (42) to generate the tree. Congruence of the major fungal lineages were compared against the kingdom-wide concatenation tree proposed by Li *et al.* (45) and visualized as a tanglegram (Supplementary Figure S3).

### Quantification of the congruence between UFCG and BUSCO trees

To quantify the power of the markers in delineating relationships from different taxonomic ranks, we measured the normalized Robinson-Foulds distances ((46); Supplementary Figure S4) to compare trees built from 758 BUSCO marker genes to the UFCG marker gene generated trees at different ranks. We grouped the 1587 species-representative genomes by their class, order, family and genus annotation and selected the groups with 30 or more genomes with identical taxonomic names for each rank (15 or more for genera). For intra-species analysis, we listed the species with 100 or more genomes from 9397 species-redundant genomes and randomly sampled 100 genomes per species. For each taxonomic group, we built trees from the concatenated alignments of 758 BUSCOs (OrthoDB v10 fungi subset), 61 UFCG markers and their subsets (41 core and 20 canonical genes) with FastTree v2.1.10 using UFCG v1.0 tree module. We calculated the Robinson-Foulds distances between UFCG trees, core gene trees and canonical gene trees against BUSCO trees using GoTree v0.4.3 (47).

## RESULTS AND DISCUSSION

### UFCG marker genes

We defined a set of 61 well-annotated and representative genes, namely UFCG marker genes (Supplementary Table S5). Determined by our computational pipeline, we included 41 core genes with 95% single-copy existence across 1,587 species-representative fungal genome assemblies. Additionally, we added the genes which have been frequently used to delineate higher-level classification of fungi (e.g. *RPB2*, *TEF1*, *TUB2* for phylum *Basidiomycota*) by fungal communities, resulting in 20 canonical genes (Supplementary Table S6).

**Figure 2.**
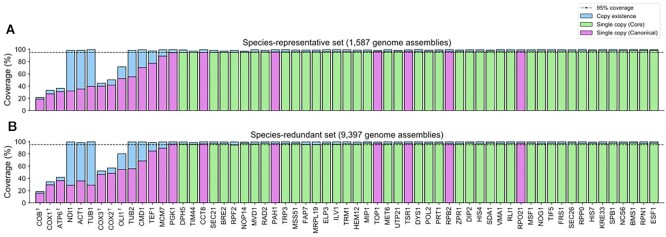
Existence coverage of 61 UFCG marker genes, represented as a proportion of fungal genome assemblies with a valid hit. (**A**) Coverage against 1587 species-representative assemblies. (**B**) Coverage against 9397 species-redundant assemblies. Presence of each marker gene against the given set of genome assemblies was identified using an AUGUSTUS-PPX search with their corresponding block profile HMMs. We then tallied the proportion of genome assemblies in which marker genes were (i) present, regardless of copy-number (blue bars) and (ii) present as single-copy (purple bars for canonical genes, green bars for core genes). Genes of mitochondrial origin (as annotated by the *Saccharomyces* genome database) were marked with a dagger (e.g. COX1^†^). Gene names are sorted by their single-copy coverage against the species-representative assemblies.

Of the 62 candidate core genes, 41 covered ≥95% of the species-representative genomes in our dataset as single-copy (Figure 2A). The remaining 21 failed the coverage threshold criterion and were rejected from the final set of core genes (Supplementary Figure S1). When extended to the 9397 species-redundant genome set, 40 of the 41 were identified as single-copy in ≥95% of the genomes (Figure 2B).

Meanwhile, only 7 out of 20 canonical genes met 95% single-copy threshold, while the main reason we speculate for this is the missing mitochondrial DNA in the genome assemblies. All but six canonical genes reported ≥98% coverage disregarding their copy numbers, in both the species-representative and the redundant set. These six genes are located on a mitochondrial genome, according to *Saccharomyces* genome database annotation (48), which most likely are universal genes that exists in ≥95% of species (49). However, 89.6% of the fungal genomes in the GenBank database are in a draft state (i.e. assembled below chromosome level) and therefore miss a certain fraction of DNA. We speculate that the mtDNA is especially affected by this since they might be deposited independently or might be rejected due to its uneven coverage in comparison to the remaining genomic DNA (50).

We defined a relatively small set of universal single-copy markers from the Swiss-Prot database with stringent single-copy existence and sequence coverage thresholds. The Swiss-Prot database covers the entire reference proteome of the species *Saccharomyces cerevisiae* and thus should contain genes that ought to be universally conserved in yeasts and other fungal species across the kingdom (51). Also, every threshold picked during the marker gene generation introduces a bias that might result in a different marker gene set. The most prominent is the single-copy existence threshold, which we set to 95% to allow fully automatic phylogenetic analysis. By lowering this threshold to 90%, 85% and 80%, we obtained 483, 829 and 1165 proteins, respectively.

### Database contents

The UFCG gene database presents a summarized list of both core and canonical marker genes we defined, as well as descriptions of individual genes with downloadable resources (Figure 1A, top). We prepared pre-trained block profile HMMs with both aligned and unaligned homologous amino acid sequences used to generate the models. Visualized MSAs are also available, constructed with the amino acid sequences extracted from 75 representative fungal species, which were implemented with MSAViewer (52). In addition, we offer direct links to the entries to external databases with corresponding annotations, including the *Saccharomyces* genome database (SGD; (48)), UniProt (35) and NCBI Conserved Domain Database (CDD; (53)).

The UFCG species database contains pre-extracted sequences of UFCG markers, ITS region, and BUSCO identified in the set of representative genome assemblies from 1587 fungal species as described (Figure 1A, bottom). Extracted sequences, metadata of originating genomes, and auxiliary run-time information were compiled into the JSON files (under field data, genome_info, run_info, respectively), which are downloadable from the database. We organized them into a sortable and searchable table, which provides the download links along with their NCBI accession numbers and taxonomic annotations. Additionally, we extracted UFCG markers, ITS region, and BUSCO sequences from 9397 species-redundant genome assemblies, which are also downloadable from the database as compressed archives.

The UFCG database will continue to be updated with enriched sequences and MSAs of marker genes and pre-extracted sequences as new NCBI GenBank releases become available.

### Phylogenetic analysis with combined sequence types

**Figure 3.**
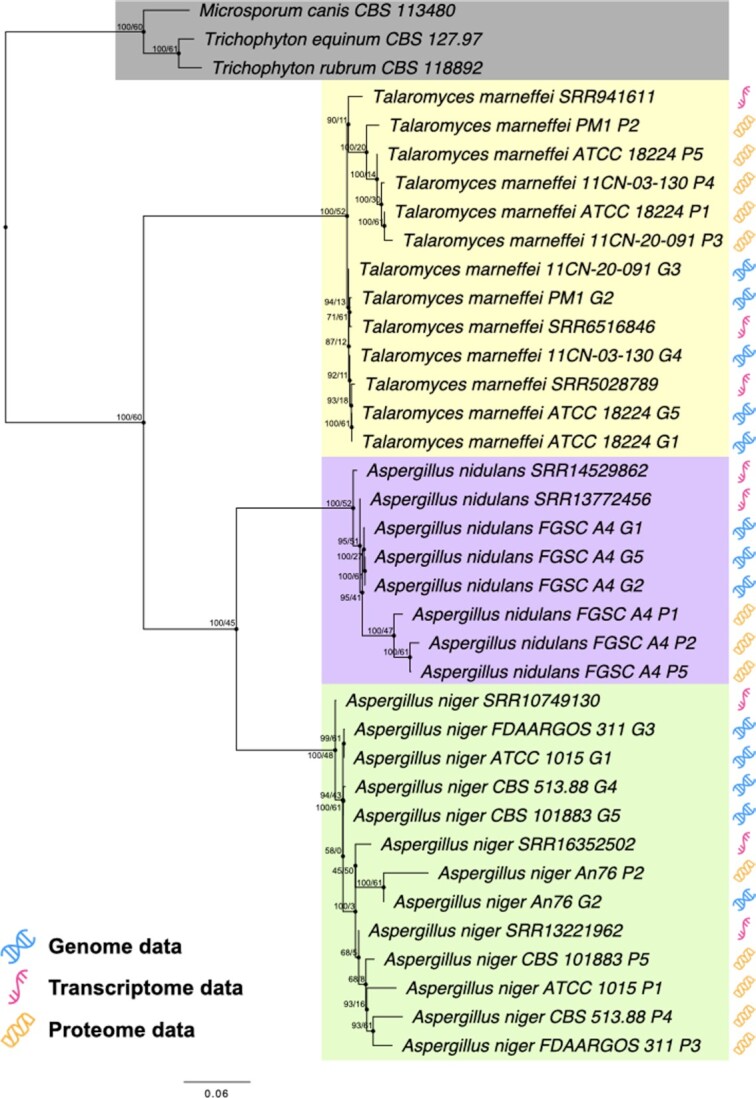
Maximum likelihood (ML) tree of the concatenated alignment of UFCG marker genes, extracted from either genomic, transcriptomic or proteomic data from 34 sequence datasets originated from three species under the order Eurotiales. As outgroup we included three species from the order Onygenales (highlighted in grey). Branches of the resulting tree were annotated by their bootstrap support and GSI values. monophyletic clades clustered by their species origin were highlighted with coloured box (yellow, *Talaromyces marneffei*; purple, *Aspergillus nidulans*; green, *Aspergillus niger*). Type of sequence origin was marked with the respective symbol (refer to the legend).

The UFCG pipeline can extract marker genes from assorted types of biological sequences, including DNA, RNA and protein. To illustrate this, we constructed a phylogenetic tree of UFCG marker genes extracted from 13 genomic, 8 transcriptomic and 13 proteomic sequences, originating from three species under the order Eurotiales (Figure 3). As shown by the topology of monophyletic clades grouped by their species origin, our gene database and pipeline successfully reconstructed the phylogenetic relationship from raw biological data in a fully automated procedure, regardless of their data types.

### Kingdom-wide phylogenetic reconstruction

One of the key advantages of the UFCG pipeline is its ability to automatically construct phylogenies from computationally detectable core genes. This is particularly useful for larger genome datasets where manual extraction of marker genes is prohibitive.

We reconstructed a kingdom-wide phylogenetic relationship of 1587 species-representative assemblies using UFCG marker genes extracted with our pipeline (Supplementary Figure S2). When compared to a previously published genome-scale fungal phylogeny built using BUSCO sequences (45), 14 out of 18 major lineages of fungi were congruent (Supplementary Figure S3). Although two incongruent pairs were observed (*Wallemiomycotina* and *Ustilagomycotina*; *Glomeromycotina* and *Mortierellomycotina*), the placement of these pairs have been contentious throughout previous studies (54,55).

### Comparison of UFCG trees with BUSCO trees across various taxonomic ranks

To test the power of UFCG markers at resolving relationships across different taxonomic ranks, we performed a deeper comparison between trees built using UFCG marker genes and 758 BUSCOs by measuring their congruence based on normalized Robinson–Foulds distance (Supplementary Figure S4). UFCG markers reconstructed phylogenies consistent with the BUSCO tree at the intra-class, intra-order, intra-family, and intra-genus levels, with 87.7%, 89.3%, 86.3% and 85.9% congruence, respectively.

Topology was not consistent at the intra-species level (10.9% congruence), however, which we speculate is due to the highly-conserved nature of the marker genes. In such cases, markers with higher resolution (e.g. nucleotide markers, genes from pan-genome analysis) can be easily trained into prediction models with the train module of our pipeline, which can be integrated with the UFCG markers for downstream phylogenetic analysis.

Additionally, we constructed separate phylogenies from the 41 core gene markers and the 20 canonical markers and performed the same comparison. Trees built from the core gene markers were significantly more congruent with the BUSCO tree compared to those built with just the canonical genes (paired-*t*, *P* = 9.57 × 10^−10^), implying the congruence of UFCG tree with BUSCO tree was predominantly derived from the core gene markers we defined rather than those canonically accepted.

Put together, we demonstrated the usefulness and consistency of our condensed and well-annotated set of marker genes, which is capable of reconstructing from genus-wide to kingdom-wide relationship of fungi.

### Concluding remarks

Our novel database of fungal marker genes and pipeline provides a robust and easy-to-use method for genome-wide phylogenetic analysis of fungi. Similar approaches for prokaryotic communities such as Genome Taxonomy Database (GTDB; (19)), AutoMLST (20) and UBCG (21) have shown the value that automatic phylogenetic analysis brings. As the first fungal core gene database with an automated phylogenetic pipeline, we expect UFCG to be of similar interest and help to tackle the challenge of genome-scale fungal phylogenetic analysis.

## DATA AVAILABILITY

The UFCG database is freely available without registration at https://ufcg.steineggerlab.com. Entire content of the database is licensed under CC BY-SA 4.0. The pipeline is implemented in Java and is available as GPLv3 licensed free open-source software at https://github.com/steineggerlab/ufcg.

## Supplementary Material

gkac894_Supplemental_File

## Notes

Present address: Jongsik Chun, CJ Bioscience, Seoul 04257, Republic of Korea.
