# Supplementary material for: UFCG: database of universal fungal core genes and pipeline for genome-wide phylogenetic analysis of fungi
Source: Nucleic Acids Res. 2022 Oct 22;51(D1):D777–84. doi: 10.1093/nar/gkac894 (PMC9825530; doi:10.1093/nar/gkac894)
Supplement: gkac894_Supplemental_File [file gkac894_supplemental_file.pdf]

Published online

UFCG: database of universal fungal core genes and pipeline for genome-wide phylogenetic analysis of fungi

Dongwook Kim[1], **Cameron L.M. Gilchrist**[2], **Jongsik Chun**[1,2,3,*], **and Martin Steinegger**[1,2,3,4,*]

[1]Interdisciplinary Program in Bioinformatics, Seoul National University, Seoul 08826, Republic of Korea, [2]School of Biological Sciences, Seoul National University, Seoul 08826, Republic of Korea, [3]Institute of Molecular Biology and Genetics, Seoul National University, Seoul 08826, Republic of Korea, [4]Artificial Intelligence Institute, Seoul National University, Seoul 08826, Republic of Korea

*To whom correspondence should be addressed. Email: jchun@snu.ac.kr, martin.steinegger@snu.ac.kr;
Present address: Jongsik Chun, CJ Bioscience, Seoul 04257, Republic of Korea

© The Author(s)
This is an Open Access article distributed under the terms of the Creative Commons Attribution Non-Commercial License (http://creativecommons.org/licenses/by-nc/2.0/uk/) which permits unrestricted non-commercial use, distribution, and reproduction in any medium, provided the original work is properly cited.

SUPPLEMENTARY MATERIALS

Tables

- Supplementary Table 1. Statistics of AUGUSTUS gene prediction of species models from 5 fungal phyla.
- Supplementary Table 2. Estimated running time of UFCG `profile` and `tree` modules.
- Supplementary Table 3. Commands and parameters for UFCG pipeline to generate the UFCG trees.
- Supplementary Table 4. Description of 34 sequences originated from 3 species under the order Eurotiales.
- Supplementary Table 5. Detailed information of 61 UFCG marker genes.
- Supplementary Table 6. List of 20 canonical marker genes.

Figures

- Supplementary Figure 1. Existence coverage of 21 candidate core marker genes below 95% single copy proportion.
- Supplementary Figure 2. Topology of the maximum likelihood tree of 1,587 genome asseblies representing fungal species.
- Supplementary Figure 3. Tanglegram comparing the topologies of two kingdom-wide tree of fungal species.
- Supplementary Figure 4. Congruence of UFCG trees with BUSCO trees.

Supplementary Table 1. Statistics of AUGUSTUS gene prediction count from 1,587 species-representative genome assemblies, using pre-trained species models from 5 fungal phyla.

Species	Phylum	Average prediction count (±SE)
Encephalitozoon cuniculi	Rozellomycota	4128.53 (±76.2312)
Conidiobolus coronatus	Entomophthoromycota	11478.4 (±194.335)
Rhizopus oryzae	Mucoromycota	**13550.8** (±215.982)
Saccharomyces cerevisiae	Ascomycota	7625.03 (±99.7421)
Cryptococcus neoformans	Basidiomycota	9009.65 (±123.441)

Supplementary Table 2. Expected running time of UFCG `profile` and `tree` modules. Benchmark was performed with Intel® Xeon® Platinum 8358 processor. Statistics of the time elapsed to run the modules are presented with benchmark details including CPU threads, number of genomes, number of replicates, and command line arguments for the pipeline.

Module	Threads	Genomes	Replicates	Command line arguments	Time elapsed (sec; ±SE)
`profile`	8	1	30	`profile -i FASTA -o OUT -t 8`	137.2 (±4.285)
`profile`	32	1	30	`profile -i FASTA -o OUT -t 32`	54.83 (±1.120)
`profile`	64	1	30	`profile -i FASTA -o OUT -t 64`	53.13 (±1.250)
`tree` (IQ-TREE)	8	30	10	`tree -i IN -l uid -o OUT -p iqtree -t 8`	692.3 (±15.52)
`tree` (IQ-TREE)	32	30	10	`tree -i IN -l uid -o OUT -p iqtree -t 32`	413.2 (±7.286)
`tree` (IQ-TREE)	64	30	10	`tree -i IN -l uid -o OUT -p iqtree -t 64`	445.1 (±6.978)
`tree` (FastTree)	8	30	10	`tree -i IN -l uid -o OUT -p fasttree -t 8`	196.3 (±2.394)
`tree` (FastTree)	32	30	10	`tree -i IN -l uid -o OUT -p fasttree -t 32`	148.9 (±3.533)
`tree` (FastTree)	64	30	10	`tree -i IN -l uid -o OUT -p fasttree -t 64`	146.6 (±3.434)

Note. Benchmarks of the `train` module is omitted since the module is a combination of `profile` module (for extraction) and `tree` module (for alignment).

Supplementary Table 3. Commands and parameters for UFCG pipeline to generate the UFCG trees from 34 Eurotiales species and 1,587 fungal assemblies

Task	Command
Marker gene extraction from genomes	`java -jar UFCG.jar profile -i FASTA -o OUT --info METADATA`
Marker gene extraction from transcriptomes	`java -jar UFCG.jar profile-rna -p 1 -i FASTQ -o OUT --info METADATA`
Marker gene extraction from proteomes	`java -jar UFCG.jar profile-pro -i FASTA -o OUT --info METADATA`
IQ-TREE generation (Eurotiales)	`java -jar UFCG.jar tree -i IN -l label -o OUT -a protein -p iqtree`
FastTree generation (Kingdom-wide, lower-level)	`java -jar UFCG.jar tree -i IN -l label -o OUT -a protein -p fasttree`

Supplementary Table 4. Description of 34 genomic, transcriptomic, and proteomic sequences retrieved from NCBI, originated from 3 species under the order Eurotiales: *Talaromyces marneffei*, *Aspergillus nidulans*, and *Aspergillus niger*.

Species	Sequence type	Label	NCBI accession
Talaromyces marneffei	Genome	Talaromyces marneffei ATCC 18224 G1	GCA_000001985.1
Talaromyces marneffei	Genome	Talaromyces marneffei PM1 G2	GCA_000750115.1
Talaromyces marneffei	Genome	Talaromyces marneffei 11CN-20-091 G3	GCA_009556855.1
Talaromyces marneffei	Genome	Talaromyces marneffei 11CN-03-130 G4	GCA_009650675.1
Talaromyces marneffei	Genome	Talaromyces marneffei ATCC 18224 G5	GCF_000001985.1
Talaromyces marneffei	Proteome	Talaromyces marneffei ATCC 18224 P1	GCA_000001985.1
Talaromyces marneffei	Proteome	Talaromyces marneffei PM1 P2	GCA_000750115.1
Talaromyces marneffei	Proteome	Talaromyces marneffei 11CN-20-091 P3	GCA_009556855.1
Talaromyces marneffei	Proteome	Talaromyces marneffei 11CN-03-130 P4	GCA_009650675.1
Talaromyces marneffei	Proteome	Talaromyces marneffei ATCC 18224 P5	GCF_000001985.1
Talaromyces marneffei	Transcriptome	Talaromyces marneffei SRR5028789	SRR5028789
Talaromyces marneffei	Transcriptome	Talaromyces marneffei SRR6516846	SRR6516846
Talaromyces marneffei	Transcriptome	Talaromyces marneffei SRR941611	SRR941611
Aspergillus nidulans	Genome	Aspergillus nidulans FGSC A4 G1	GCA_000011425.1
Aspergillus nidulans	Genome	Aspergillus nidulans FGSC A4 G2	GCA_000149205.2
Aspergillus nidulans	Genome	Aspergillus nidulans FGSC A4 G5	GCF_000149205.2
Aspergillus nidulans	Proteome	Aspergillus nidulans FGSC A4 P1	GCA_000011425.1
Aspergillus nidulans	Proteome	Aspergillus nidulans FGSC A4 P2	GCA_000149205.2
Aspergillus nidulans	Proteome	Aspergillus nidulans FGSC A4 P5	GCF_000149205.2
Aspergillus nidulans	Transcriptome	Aspergillus nidulans SRR13772456	SRR13772456
Aspergillus nidulans	Transcriptome	Aspergillus nidulans SRR14529862	SRR14529862
Aspergillus niger	Genome	Aspergillus niger ATCC 1015 G1	GCA_000230395.2
Aspergillus niger	Genome	Aspergillus niger An76 G2	GCA_001515345.1
Aspergillus niger	Genome	Aspergillus niger FDAARGOS_311 G3	GCA_002211485.2
Aspergillus niger	Genome	Aspergillus niger CBS 513.88 G4	GCF_000002855.3
Aspergillus niger	Genome	Aspergillus niger CBS 101883 G5	GCF_003184595.1
Aspergillus niger	Proteome	Aspergillus niger ATCC 1015 P1	GCA_000230395.2
Aspergillus niger	Proteome	Aspergillus niger An76 P2	GCA_001515345.1
Aspergillus niger	Proteome	Aspergillus niger FDAARGOS_311 P3	GCA_002211485.2
Aspergillus niger	Proteome	Aspergillus niger CBS 513.88 P4	GCF_000002855.3
Aspergillus niger	Proteome	Aspergillus niger CBS 101883 P5	GCF_003184595.1
Aspergillus niger	Transcriptome	Aspergillus niger SRR10749130	SRR10749130
Aspergillus niger	Transcriptome	Aspergillus niger SRR13221962	SRR13221962
Aspergillus niger	Transcriptome	Aspergillus niger SRR16352502	SRR16352502

Supplementary Table 5. Detailed information of 61 UFCG marker genes. Genes are labeled by their *Saccharomyces* genome database (SGD) names.

Gene	Type	SGD ID	UniProt ID	CDD ID	COG*	Function
ACT1	Canonical	YFL039C	P60010	KOG0676	Z	γ-Actin
ATP6	Canonical	Q0085	P00854	KOG4665	C	F_1F_0 ATP synthase subunit 6
BMS1	Core	YPL217C	Q08965	KOG1951	J	Ribosome biogenesis protein
BRE2	Core	YLR015W	P43132	KOG2626	B/K	COMPASS component
CCT8	Canonical	YJL008C	P47079	KOG0362	O	Chaperonin-containing T-complex subunit θ
CMD1	Canonical	YBR109C	P06787	KOG0027	T	Calmodulin
COB	Canonical	Q0105	P00163	KOG4663	C	Cytochrome b
COX1	Canonical	Q0045	P00401	KOG4769	C	Cytochrome c oxidase subunit 1
COX2	Canonical	Q0250	P00410	KOG4767	C	Cytochrome c oxidase subunit 2
COX3	Canonical	Q0275	P00420	KOG4664	C	Cytochrome c oxidase subunit 3
DIP2	Core	YLR129W	Q12220	KOG0306	A	U3 small nucleolar RNA-associated protein 12
DPH5	Core	YLR172C	P32469	KOG3123	J	Diphthine methyl ester synthase
DYS1	Core	YHR068W	P38791	KOG2924	O	Deoxyhypusine synthase
ELP3	Core	YPL086C	Q02908	KOG2535	B/K	Elongator complex protein 3
ESF1	Core	YDR365C	Q06344	KOG2318	S	Pre-rRNA-processing protein
FAP7	Core	YDL166C	Q12055	KOG3347	F	Adenylate kinase isoenzyme 6 homolog
FRS1	Core	YLR060W	P15624	KOG2472	J	Phenylalanine–tRNA ligase beta subunit
HEM12	Core	YDR047W	P32347	KOG2872	H	Uroporphyrinogen decarboxylase
HIS4	Core	YCL030C	P00815	KOG2697	E	Histidine biosynthesis trifunctional protein
HIS7	Core	YBR248C	P33734	KOG0623	E	Imidazole glycerol phosphate synthase
ILV1	Core	YER086W	P00927	KOG1250	E	Threonine dehydratase
KRE33	Core	YNL132W	P53914	KOG2036	R	RNA cytidine acetyltransferase
MCM7	Canonical	YBR202W	P38132	KOG0482	L	Mini-chromosome maintenance complex subunit
MET6	Core	YER091C	P05694	KOG2263	E	5-methyltetrahydropteroyltriglutamate–homocysteine methyltransferase
MIP1	Core	YOR330C	P15801	KOG3657	L	DNA polymerase γ
MRPL19	Core	YNL185C	P53875	KOG3257	J	54S ribosomal protein L19
MSF1	Core	YPR047W	P08425	KOG2783	J	Phenylalanine–tRNA ligase
MSS51	Core	YLR203C	P32335	-	O	Mitochondrial splicing suppressor protein 51
MVD1	Core	YNR043W	P32377	KOG2833	I	Diphosphomevalonate decarboxylase
NCS6	Core	YGL211W	P53088	KOG2840	R	Cytoplasmic tRNA 2-thiolation protein 1
NDI1	Canonical	YML120C	P32340	KOG2495	C	NADH-ubiquinone reductase
NOG1	Core	YPL093W	Q02892	KOG1490	R	Nucleolar GTP-binding protein 1
NOP14	Core	YDL148C	Q99207	KOG2147	J	Nucleolar complex protein 14
OLI1	Canonical	Q0130	P61829	KOG3025	C	F_1F_0 ATP synthase subunit 9
PAH1	Canonical	YMR165C	P32567	KOG2116	N/I	Phosphatidate phosphatase
PGK1	Canonical	YCR012W	P00560	KOG1367	G	Phosphoglycerate kinase
POL2	Core	YNL262W	P21951	KOG1798	L	DNA polymerase epsilon catalytic subunit A
PRT1	Core	YOR361C	P06103	KOG2314	J	Eukaryotic translation initiation factor 3 subunit B
RAD2	Core	YGR258C	P07276	KOG2520	L	DNA repair protein
RLI1	Core	YDR091C	Q03195	KOG0063	A	Translation initiation factor
RPB2	Canonical	YOR151C	P08518	KOG0214	K	DNA-directed RNA polymerase II core subunit
RPF2	Core	YKR081C	P36160	KOG3031	J	Ribosome biogenesis protein
RPN1	Core	YHR027C	P38764	KOG2005	O	26S proteasome regulatory subunit
RPO21	Canonical	YDL140C	P04050	KOG0260	K	DNA-directed RNA polymerase II core subunit
RPP0	Core	YLR340W	P05317	KOG0815	J	60S acidic ribosomal protein P0
SDA1	Core	YGR245C	P53313	KOG2229	D/Z	Severe depolymerization of actin protein 1
SEC21	Core	YNL287W	P32074	KOG1078	U	Coatomer subunit gamma
SEC26	Core	YDR238C	P41810	KOG1058	U	Coatomer subunit beta
SPB1	Core	YCL054W	P25582	KOG1098	A/R	27S pre-rRNA (guanosine$_{2922}$-2'-O)-methyltransferase
TEF1	Canonical	YPR080W	P02994	KOG0052	J	Translation elongation factor EF-1α
TIF5	Core	YPR041W	P38431	KOG2767	J	Eukaryotic translation initiation factor 5
TIM44	Core	YIL022W	Q01852	KOG2580	U	Mitochondrial import inner membrane translocase subunit
TOP1	Canonical	YOL006C	P04786	KOG0981	L	DNA topoisomerase 1
TRM1	Core	YDR120C	P15565	KOG1253	J	tRNA (guanine$_{26}$-N$_2$)-dimethyltransferase
TRP3	Core	YKL211C	P00937	KOG0026	E	Multifunctional tryptophan biosynthesis protein
TSR1	Canonical	YDL060W	Q07381	KOG1980	S	Ribosome maturation factor
TUB1	Canonical	YML085C	P09733	KOG1376	Z	α-tubulin
TUB2	Canonical	YFL037W	P02557	KOG1375	Z	β-tubulin
UTP21	Core	YLR409C	Q06078	KOG1539	R	U3 small nucleolar RNA-associated protein 21
VMA1	Core	YDL185W	P17255	KOG1540	H	V-type proton ATPase catalytic subunit A
ZPR1	Core	YGR211W	P53303	KOG2703	R	Zinc finger protein

*COG, clusters of orthologous group: A, RNA processing and modification; B, Chromatin Structure and dynamics; C, Energy production and conversion; D, Cell cycle control and mitosis; E, Amino Acid metabolis and transport; F, Nucleotide metabolism and transport; G, Carbohydrate metabolism and transport; H, Coenzyme metabolis; I, Lipid metabolism; J, Tranlsation; K, Transcription; L, Replication and repair; N, Cell motility; O, Post-translational modification, protein turnover, chaperone functions; T, Signal Transduction; U, Intracellular trafficing and secretion; Z, Cytoskeleton; R, General Functional Prediction only; S, Function Unknown.

Supplementary Table 6. List of 20 canonical marker genes with example fungal taxa with phylogenetic analysis using the markers (see also 1, 2).

Gene	Aliases	Example taxa	References
ACT1	*ACT*	*Cryptococcus*, *Glomeromycota*	3, 4
ATP6	-	*Boteales*, *Agaricus*	5, 6
CCT8	*TCP1θ*	*Aspergillus*, *Saccharomyces*	7, 8
CMD1	*CAL, CaM*	*Eurotiales*, *Penicillium*	9, 10
COB	-	*Aspergillus*, *Glomeromycota*	11, 12
COX1	-	*Pezizomycotina*, *Glomeromycota*	12, 13
COX2	-	*Peronosporomycetes*	14
COX3	-	*Boteales*	5
MCM7	*CDC47*	*Ascomycota*, *Kickxellomycotina*	15, 16
NDI1	*NAD1-6*	*Beauveria*, *Glomeromycota*	12, 17
OLI1	*mtATP9*	*Beauveria*, *Glomeromycota*	12, 17
PAH1	*LNS2*	*Pucciniomycota*	1
PGK1	*PGK*	*Fusarium*, *Penicillium*	1, 18
RPB2	-	*Ascomycota*, *Basidiomycota*	19, 20
RPO21	*RPB1*	*Inocybe*, *Zygomycota*	21, 22
TEF1	*TEF1α*	*Basidiomycota*, *Zygomycota*	19, 22
TOP1	-	*Fusarium*, *Penicillium*	1, 23
TSR1	-	*Kickxellomycotina*	15
TUB1	-	*Microsporidia*	24
TUB2	*BenA*	*Basidiomycota*, *Microsporidia*	24, 25

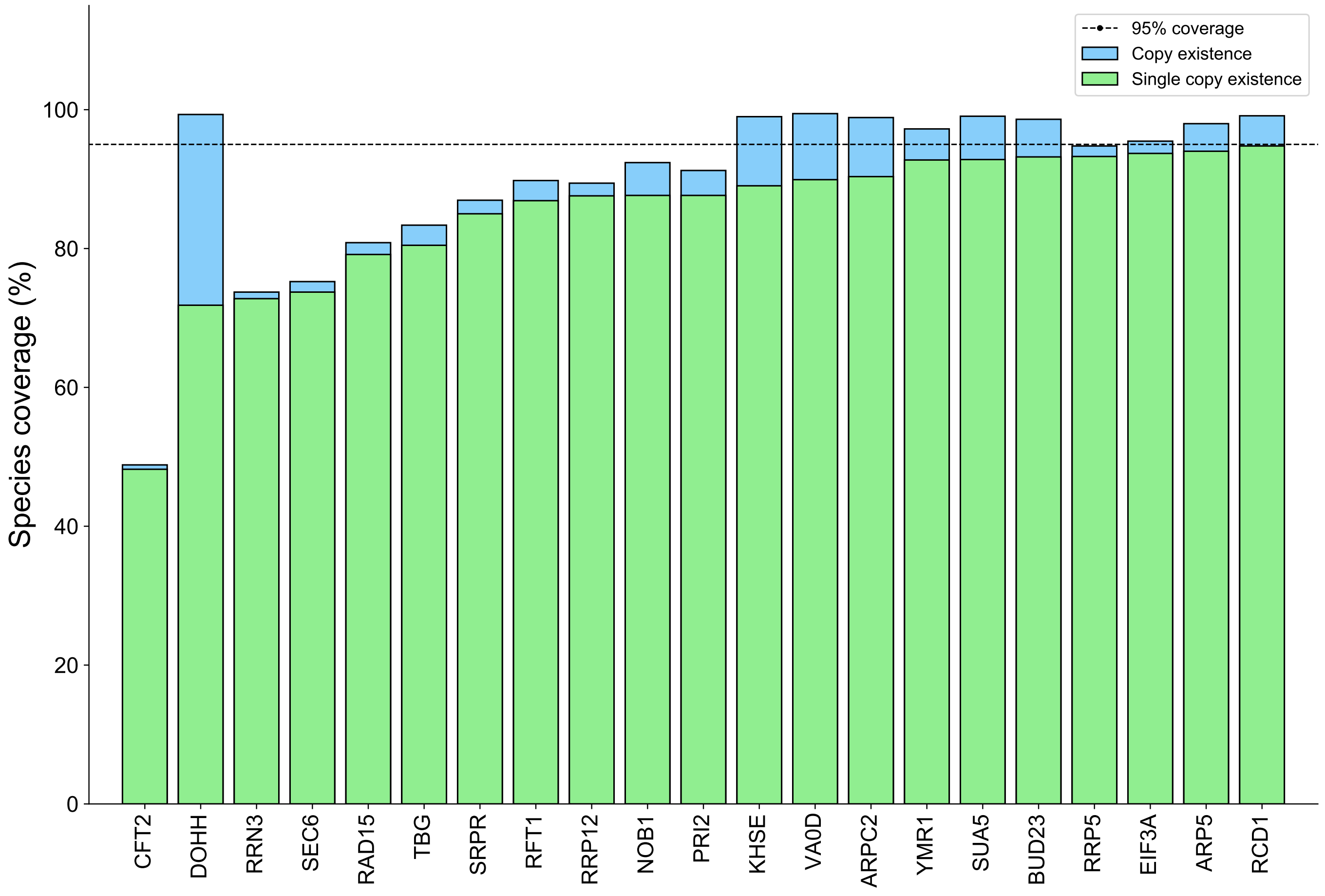

Supplementary Figure 1. Existence coverage of 21 candidate core marker genes, which failed to achieve 95% single copy proportion of covered entries among the 1,587 genome assemblies representing fungal species.

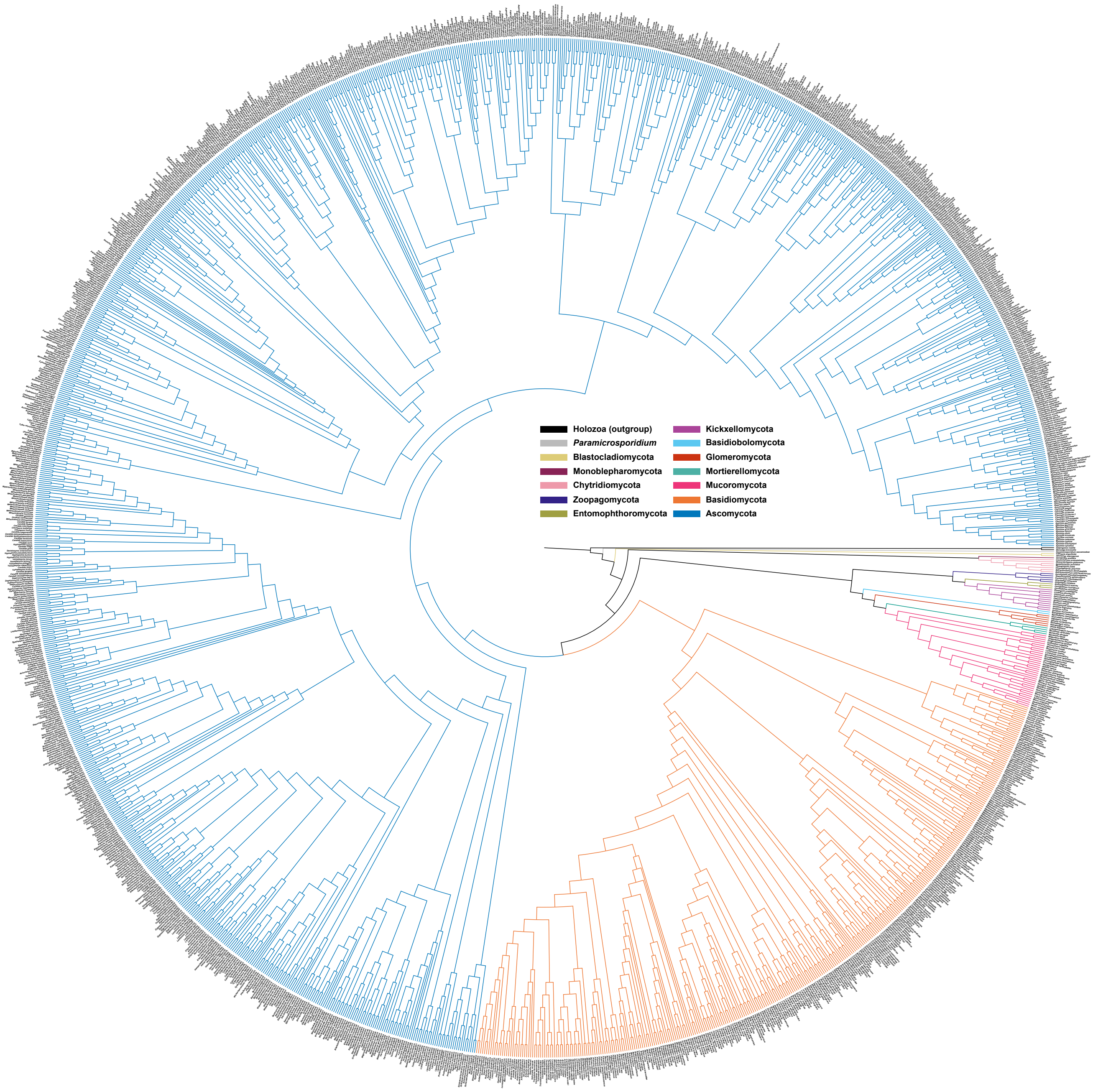

Supplementary Figure 2. Topology of the maximum likelihood tree of 1,587 genome assemblies representing fungal species. Tree was generated from the concatenated amino acid sequence alignment of 61 UFCG marker genes, using FastTree v2.1.10. Branches are coloured based on their phylum (refer to the legend).

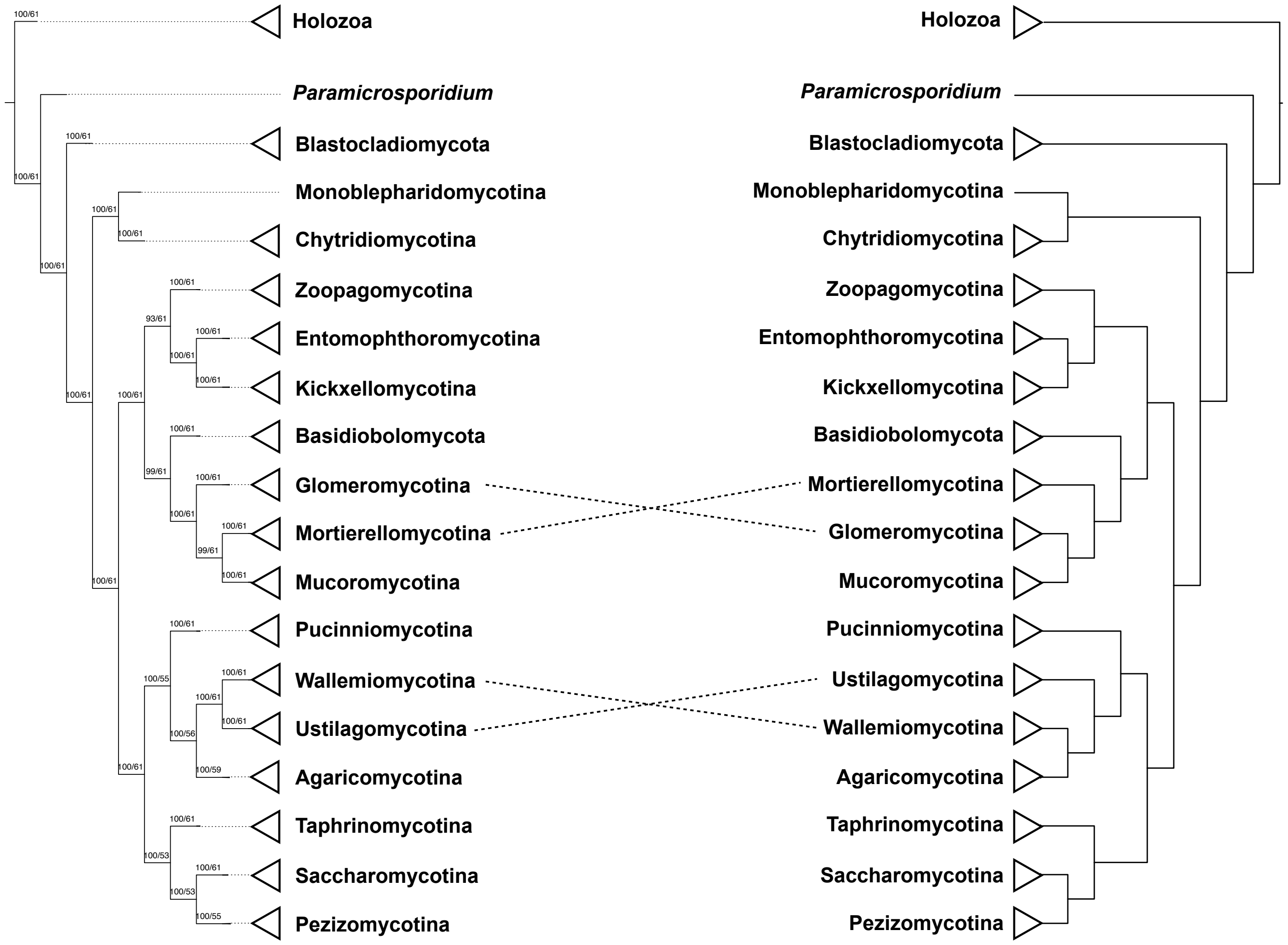

Supplementary Figure 3. Tanglegram comparing the topologies of two kingdom-wide tree of fungal species: Left, UFCG marker gene concatenation tree; Right, BUSCO concatenation tree presented by Li, et al. (26). Branches of UFCG trees were annotated by their bootstrap support and gene support index (GSI) values. Discrepancies between the trees were visualised by dotted lines connecting the corresponding clades.

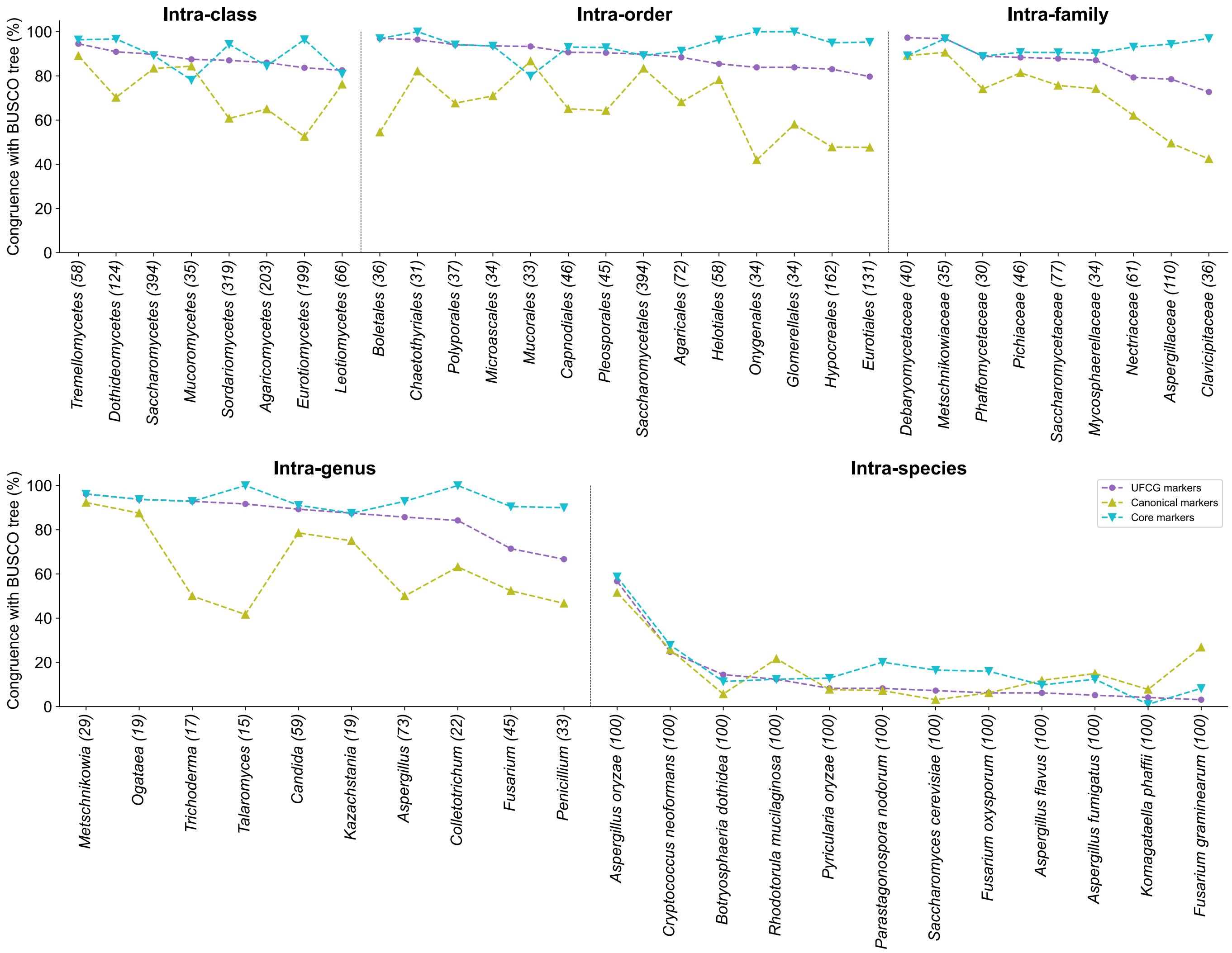

Supplementary Figure 4. Congruence of the concatenation trees using 61 UFCG markers and subset of the markers (20 canonical, 41 core) with 758 BUSCO concatenation tree, based on normalised Robinson-Foulds distance. Percentage of the congruence between tree T_1 and T_2 was defined as $(1 - \frac{RF(T_1,T_2)}{2n-6}) \times 100\%$, where $RF(T_1,T_2)$ indicates the Robinson-Foulds distance between tree T_1 and T_2, and n is the number of leaf nodes of the tree. Fungal genomes were grouped with their taxonomic name annotations with different ranks, resulted in 8 classses, 14 orders, 9 families, 10 genera, and 12 species. Data points are plotted in dashed lines (Purple dots, 61 UFCG markers; Olive triangle, 20 canonical markers; Cyan inverted triangles, 41 core markers), grouped by their taxonomic rank and sorted by the congruence of UFCG marker trees in descending order. X-axis was labeled with the taxonomic name of each group, marked with the number of genomes included in parentheses.

REFERENCES

1. Stielow, J. B. et al (2015) One fungus, which genes? Development and assessment of universal primers for potential secondary fungal DNA barcodes. *Pers.: Mol. Phylogeny Evol. Fungi,* **35**, 242–263.
2. Lücking, R. et al (2020) Unambiguous identification of fungi: where do we stand and how accurate and precise is fungal DNA barcoding?. *IMA Fungus,* **11**, 14.
3. Cox, G., Rude, T., Dykstra, C. and Perfect, J. (1995) The actin gene from Cryptococcus neoformans: structure and phylogenetic analysis. *J. Med. Vet. Mycol.,* **33**, 261–266.
4. Helgason, T., Watson, I. J. and Young, J. P. W. (2003) Phylogeny of the Glomerales and Diversisporales (Fungi: Glomeromycota) from actin and elongation factor 1-alpha sequences. *FEMS Microbiol. Lett.,* **229**, 127–132.
5. Kretzer, A. M. and Bruns, T. D. (1999) Use of atp6 in fungal phylogenetics: an example from the Boletales. *Mol. Phylogenet. Evol.,* **13**, 483–492.
6. Robison, M. M., Chiang, B. and Horgen, P. A. (2001) A phylogeny of the genus Agaricus based on mitochondrial atp 6 sequences. *Mycologia,* **93**, 30–37.
7. Kocsubé, S. et al (2016) Aspergillus is monophyletic: evidence from multiple gene phylogenies and extrolites profiles. *Stud. Mycol.,* **85**, 91–105.
8. Stoldt, V. et al (1996) The Cct eukaryotic chaperonin subunits of Saccharomyces cerevisiae and other yeasts. *Yeast,* **12**, 523–529.
9. Houbraken, J. et al (2020) Classification of Aspergillus, Penicillium, Talaromyces and related genera (Eurotiales): An overview of families, genera, subgenera, sections, series and species. *Stud. Mycol.,* **96**, 141–153.
10. Wang, L. and Zhuang, W.-Y. (2007) Phylogenetic analyses of penicillia based on partial calmodulin gene sequences. *BioSystems,* **88**, 113–126.
11. Wang, L., Yokoyama, K., Miyaji, M. and Nishimura, K. (1998) The identification and phylogenetic relationship of pathogenic species of Aspergillus based on the mitochondrial cytochrome b gene. *Med. Mycol.,* **36**, 153–164.
12. Nadimi, M., Daubois, L. and Hijri, M. (2016) Mitochondrial comparative genomics and phylogenetic signal assessment of mtDNA among arbuscular mycorrhizal fungi. *Mol. Phylogenet. Evol.,* **98**, 74–83.
13. Damon, C. et al (2010) Performance of the COX1 gene as a marker for the study of metabolically active Pezizomycotina and Agaricomycetes fungal communities from the analysis of soil RNA. *FEMS Microbiol. Ecol.,* **74**, 693–705.
14. Hudspeth, D. S., Nadler, S. A. and Hudspeth, M. E. (2000) A COX2 molecular phylogeny of the Peronosporomycetes. *Mycologia,* **92**, 674–684.
15. Tretter, E. D., Johnson, E., Wang, Y., Kandel, P. and White, M. M. (2013) Examining new phylogenetic markers to uncover the evolutionary history of early-diverging fungi: comparing MCM7, TSR1 and rRNA genes for single-and multi-gene analyses of the Kickxellomycotina. *Pers.: Mol. Phylogeny Evol. Fungi,* **30**, 106–125.
16. Raja, H., Schoch, C. L., Hustad, V., Shearer, C. and Miller, A. (2011) Testing the phylogenetic utility of MCM7 in the Ascomycota. *MycoKeys,* **1**, 63–94.
17. Ghikas, D. V., Kouvelis, V. N. and Typas, M. A. (2010) Phylogenetic and biogeographic implications inferred by mitochondrial intergenic region analyses and ITS1-5.8 S-ITS2 of the entomopathogenic fungi Beauveria bassiana and B. brongniartii. *BMC Microbiol.,* **10**, 174.
18. Al-Hatmi, A. M. et al (2016) Evaluation of two novel barcodes for species recognition of opportunistic pathogens in Fusarium. *Fungal Biol.,* **120**, 231–245.
19. Matheny, P. B. et al (2007) Contributions of rpb2 and tef1 to the phylogeny of mushrooms and allies (Basidiomycota, Fungi). *Mol. Phylogenet. Evol.,* **43**, 430–451.
20. Liu, Y. J., Whelen, S. and Hall, B. D. (1999) Phylogenetic relationships among ascomycetes: evidence from an RNA polymerse II subunit.. *Mol. Biol. Evol.,* **16**, 1799–1808.
21. Matheny, P. B., Liu, Y. J., Ammirati, J. F. and Hall, B. D. (2002) Using RPB1 sequences to improve phylogenetic inference among mushrooms (Inocybe, Agaricales). *Am. J. Bot.,* **89**, 688–698.
22. Tanabe, Y., Saikawa, M., Watanabe, M. M. and Sugiyama, J. (2004) Molecular phylogeny of Zygomycota based on EF-1α and RPB1 sequences: limitations and utility of alternative markers to rDNA. *Mol. Phylogenet. Evol.,* **30**, 438–449.
23. Kusari, S., Zühlke, S. and Spiteller, M. (2011) Effect of artificial reconstitution of the interaction between the plant Camptotheca acuminata and the fungal endophyte Fusarium solani on camptothecin biosynthesis. *J. Nat. Prod.,* **74**, 764–775.
24. Keeling, P. J. (2003) Congruent evidence from α-tubulin and β-tubulin gene phylogenies for a zygomycete origin of microsporidia. *Fungal Genet. Biol.,* **38**, 298–309.
25. Begerow, D., Beate, J. and Oberwinkler, F. (2004) Evolutionary relationships among β-tubulin gene sequences of basidiomycetous fungi. *Mycol. Res.,* **108**, 1257–1263.
26. Li, Y. et al (2021) A genome-scale phylogeny of the kingdom Fungi. *Curr. Biol.,* **31**, 1653–1665.
